# Targeted *in situ* metatranscriptomics for selected taxa from mesophilic and thermophilic biogas plants

**DOI:** 10.1111/1751-7915.12982

**Published:** 2017-12-04

**Authors:** Yvonne Stolze, Andreas Bremges, Irena Maus, Alfred Pühler, Alexander Sczyrba, Andreas Schlüter

**Affiliations:** ^1^ Center for Biotechnology ‐ CeBiTec Bielefeld University Universitätsstraße 27 33615 Bielefeld Germany; ^2^ Faculty of Technology Bielefeld University Universitätsstraße 25 33615 Bielefeld Germany

## Abstract

Biogas production is performed anaerobically by complex microbial communities with key species driving the process. Hence, analyses of their *in situ* activities are crucial to understand the process. In a previous study, metagenome sequencing and subsequent genome binning for different production‐scale biogas plants (BGPs) resulted in four genome bins of special interest, assigned to the phyla *Thermotogae*,* Fusobacteria*,* Spirochaetes* and *Cloacimonetes*, respectively, that were genetically analysed. In this study, metatranscriptome sequencing of the same BGP samples was conducted, enabling *in situ* transcriptional activity determination of these genome bins. For this, mapping of metatranscriptome reads on genome bin sequences was performed providing transcripts per million (TPM) values for each gene. This approach revealed an active sugar‐based metabolism of the *Thermotogae* and *Spirochaetes* bins and an active amino acid‐based metabolism of the *Fusobacteria* and *Cloacimonetes* bins. The data also hint at syntrophic associations of the four corresponding species with methanogenic *Archaea*.

## Introduction

The availability of fossil fuels is limited while the demand for energy increases steadily in the private and industrial sector, due to factors like affluence and population growth (Malik *et al*., 2016). Additionally, the consumption of natural gas and especially petroleum and coal leads to large amounts of greenhouse gas (GHG) emissions, mainly CO_2_ and CH_4_, implicating climate change and global warming (Liao *et al*., 2016; Malik *et al*., 2016).

Fuels produced from renewable sources are increasingly important alternatives to provide environmental‐friendly energy (Weiland, 2010; Zhang *et al*., 2016). Biogas is one of these important alternatives, which is produced by anaerobic digestion (AD) and mostly consists of CH_4_ with smaller proportions of CO_2_ and other impurities (Ge *et al*., 2016). In industrial‐sized biogas plants (BGPs), biogas production and usage take place under controlled conditions as they are connected to combined heat and power (CHP) systems where biogas is combusted to provide electricity and heat. For biogas production, a wide variety of substrates, e.g. energy crops like maize and organic household, industrial, slaughterhouse and agricultural wastes, can be used mostly in mixtures (Weiland, 2010; Mao *et al*., 2015; Ge *et al*., 2016; Zhang *et al*., 2016). Aside from substrate input, main differences in the setup of biogas plants concern the process temperature, as they can be run at mesophilic (35–42°C) and thermophilic (45–60°C) conditions. While mesophilic biogas processes are more stable and feature lower energy demand, biomass turnover is faster and methane yield is higher in thermophilic BGPs (Weiss *et al*., 2008; Weiland, 2010; Mao *et al*., 2015; Ruile *et al*., 2015).

The anaerobic digestion of biomass into methane can formally be subdivided into four phases, namely hydrolysis, acidogenesis, acetogenesis and methanogenesis. Within these phases, specialized groups of *Bacteria* and *Archaea* are responsible for the degradation of their respective substrates and are sometimes closely linked by syntrophic interactions. Hydrolysis is the first step in which bacteria break down complex polymers, like carbohydrates, lipids and proteins, into mono‐ and oligomers that are subsequently fermented by acidogenic and acetogenic bacteria to volatile fatty acids, alcohols, acetate, H_2_ and CO_2_. The last step, in which acetate (acetoclastic) or CO_2_ and H_2_ (hydrogenotrophic) are converted into methane, is solely performed by methanogenic *Archaea* (Weiland, 2010; Mao *et al*., 2015; Campanaro *et al*., 2016). Within the last years, the complex biogas‐producing microbial communities have been studied with regard to their members and their respective functions, but are still not fully understood. Culture‐dependent approaches include isolation, culturing, phenotypic analyses and sequencing of single community members (e.g. Maus *et al*., 2016). However, the culturing approach is limited as not all *Bacteria* and *Archaea* can be cultured and do not necessarily represent dominant and therefore functionally important members of the community. Thus, culture‐independent approaches, like metagenome and metatranscriptome sequencing, are frequently used to access the communities’ functional potential and determine transcriptional activity (e.g. Zakrzewski *et al*., 2012; Eikmeyer *et al*., 2013; Kovács *et al*., 2013; Bremges *et al*., 2015; Stolze *et al*., 2015, 2016). However, it is important to determine *in situ* functions of single microorganisms within the fermenters to better understand the process and, in the long run, enable optimization of the biogas production process. Therefore, metagenome assembly and subsequent binning of assembled contigs into genome bins are used as an approach to access single genomes within the microbial community, circumventing the need of cultivation (Kunath *et al*., 2017; Sczyrba *et al*., 2017). In this approach, species genomes are reconstructed from metagenome data sets representing a microbial community, enabling the reconstruction of their metabolic potential and abundance determination by mapping back metagenome reads on the respective genome bins (Mande *et al*., 2012; Sharpton, 2014; Sangwan *et al*., 2016). Binning has previously been used on biogas communities from laboratory‐ and production‐scale biogas production reactors, resulting in reconstruction of unknown species (Campanaro *et al*., 2016; Stolze *et al*., 2016; Treu *et al*., 2016; Xia *et al*., 2016; Kougias *et al*., 2017). Still, a study on the actual role within the community and these species’ *in situ* metabolic transcriptional activity within their respective habitats is missing.

In this study, we determined the *in situ* transcriptional activity of four genome bins originating from deeply sequenced metagenomes obtained from mesophilic and thermophilic agricultural biogas systems using corresponding metatranscriptome data. The four genome bins, of which three are novel and uncharacterized, represent species of the bacterial phyla *Thermotogae*,* Fusobacteria*,* Spirochaetes* and *Cloacimonetes* (WWE1) respectively. They have been previously selected due to their taxonomic affiliation and genomically characterized (Stolze *et al*., 2016). Analyses on the four species represented by the genome bins gave insights into their actual transcriptional activities and showed their respective metabolism and role within their habitats.

## Results and Discussion

### Metatranscriptome sequencing and read mapping

In this study, the actual *in situ* transcriptional activity of the species represented by four distinct genome bins was analysed to determine their transcriptional profiles and with this their roles within the biogas production process. For this purpose, RNA was extracted simultaneously from the same samples as the metagenomic DNA was derived from and metatranscriptome sequencing was performed in duplicates. In total, 900 million reads (137 Gbp; Table 1) were generated for one mesophilic and one thermophilic BGP. For the evaluation of transcriptional activities, the metatranscriptome reads from the BGPs were mapped on selected genome bins, counted and normalized on gene length and data set size resulting in transcripts per million (TPM) values.

**Table 1:** Metatranscriptome sequencing results

Biogas plant sample	Technical replicate	No. of reads	No. of bases
Mesophilic	1	261 433 302	39 214 995 300
	2	258 702 414	38 805 362 100
Thermophilic	1	161 677 326	24 251 598 900
	2	233 914 040	35 087 106 000
Total	–	915 727,082	137 359 062 300

To determine whether the postulated metabolic potentials of the four bins correlate with their transcriptomic activities and whether relevant genes show high transcriptional rates under *in situ* conditions, their TPM values were further analysed. Next to the general evaluation of the 25 most highly transcribed genes of the genome bins (see Table S1, S2, S3 and S4), analyses of the bins’ activity in carbohydrate degradation, fermentation pathways and syntrophic associations were performed in depth by determining TPMs for respective meaningful genes. To enable a direct examination of high, moderate or low transcriptional activity of these genes, their TPM values were assigned to categories, ranging from 1 (within the lowest 10%) up to 10 (within the top 10% transcripts). Table 2 lists TPM values and respective categories for genes encoding carbohydrate‐active enzymes, chosen by their relevance in anaerobic digestion according to Vanwonterghem *et al*. (2016). Table 3 shows those for enzymes involved in fermentation pathways. It was recently suggested that all four genome bins may be syntrophically associated with methanogenic *Archaea* (Stolze *et al*., 2016). Therefore, transcriptional activities of genes encoding enzymes potentially involved in syntrophy, according to Worm *et al*. (2014), were analysed regarding presence and transcriptional activity for each genome bin. The results are shown in Table 4. For better clarity, in all three Tables, only those genes are shown that were found in at least one of the genome bins (the complete tables are represented in the Tables S1, S2, S3, S4, S5, S6 and S7). In the following chapters, each genome bin is discussed regarding these analyses.

**Table 2:** Glycosyl hydrolase (GH) families relevant for anaerobic digestion according to Vanwonterghem *et al*. (2016) and their respective transcript per million (TPM) values and transcriptional categories for each of the four analysed genome bins. The categories range from 0 (no transcription) and 1 (lowest 10% of transcripts) to 10 (top 10% transcripts). n.d.: not detected

Enzymes	Glycoside	*Thermotogae* bin		*Fusobacteria* bin		*Spirochaetes* bin		*Cloacimonetes* bin	
Enzyme type	Hydrolase family	TPM	Category	TPM	Category	TPM	Category	TPM	Category
Endo – and exo‐1,4‐β‐D‐glucanase (cellulase)	GH5	3.639	3	n.d.	–	n.d.	–	n.d.	–
Hemicellulose	GH16	4.340	3	n.d.	–	n.d.	–	0	0
	GH28	2.167	2	n.d.	–	0.023	2	n.d.	–
	GH53	41.236	8	n.d.	–	n.d.	–	n.d.	–
	GH115	n.d.	–	n.d.	–	0.132	6	n.d.	–
	GH76	n.d	–	n.d	–	0.018	1	n.d	–
Starch and glycogen Hydrolase	GH13	3.958	3	1.574	6	0.129	6	0.007	2
	GH77	n.d.	–	9.716	10	0.102	6	0	0
	GH57	35.201	8	3.788	8	0.165	7	0.021	5
Lysozyme, chitinase (cell wall degradation)	GH18	6.172	3	n.d.	–	n.d.	–	0.021	5
	GH23	20.029	6	0.655	3	n.d.	–	0.063	8
	GH73	n.d.	–	n.d	–	n.d	–	n.d	–
Glycosidase (hydrolysis of single sugar residues from non–reducing ends)	GH1	0.822	1	n.d.	–	0.074	4	n.d.	–
	GH2	38.604	8	n.d.	–	0.107	6	n.d.	–
	GH3	3.022	2	n.d.	–	0.142	6	n.d.	–
						0.069	4		
	GH4	22.249	6	n.d.	–	0.019	2	n.d.	–
		144.144	10						
	GH38	n.d.	–	n.d.	–	n.d.	–	0.004	1
	GH51	3.355	3	n.d	–	0.113	6	n.d	–
Oligosaccharide phosphorylase	GH130	n.d.	–	n.d.	–	n.d.	–	0.002	1

**Table 3:** Fermentation pathway proteins and their respective transcript per million (TPM) values and transcriptional categories for each of the four analysed genome bins. The categories range from 0 (no transcription) and 1 (lowest 10% of transcripts) to 10 (top 10% transcripts) n.d.: not detected

Fermentation type	Fermentation pathway	Enzyme	Interpro number	*Thermotogae* bin		*Fusobacteria* bin		*Spirochaetes* bin		*Cloacimonetes* bin	
				TPM	Category	TPM	Category	TPM	Category	TPM	Category
Propionic acid fermentation	Acrylyl‐CoA pathway	CoA‐transferase (EC 2.8.3.1)	IPR003702	n.d.	–	n.d.	–	n.d.	–	n.d.	–
		Lactoyl‐CoA dehydratase dehydratase	IPR010327	n.d	–	n.d.	–	0.065	3	0.009	2
		Acyl‐CoA dehydrogenase (E.C. 1.3.99.3)	IPR034179	n.d.	–	0.052	1	n.d.	–	n.d.	–
			IPR034180								
	Methylmalonyl‐ CoA pathway	Pyruvate carboxylase (EC 6.4.1.1)	IPR005930	n.d.	–	n.d.	–	n.d.	–	n.d.	–
		Malate dehydrogenase (EC 1.1.1.37)	IPR001252	25.110	7	1.308	5	n.d.	–	0.035	7
			IPR023958								
			IPR011275								
		Fumarate hydratase (EC 4.2.1.2)	IPR018951	18.872	6	0.982	4	0.13	6	0.0104	3
			IPR011167								
		Fumarate reductase (EC 1.3.5.4)	IPR005884	n.d.	–	n.d.	–	n.d.	–	n.d.	–
		Succinyl‐CoA synthetase (EC 6.2.1.4; EC 6.2.1.5)	IPR034722	n.d.	–	n.d.	–	n.d.	–	n.d.	–
			IPR005809								
			IPR005810								
		Methylmalonyl‐CoA mutase (5.4.99.2)	IPR004608	n.d.	–	0.021	1	n.d.	–	n.d.	–
			IPR024067								
		Methylmalonyl‐CoA epimerase (EC 5.1.99.1)	IPR017515	38.108	8	n.d.	–	n.d.	–	n.d.	–
		Methylmalonyl‐CoA decarboxylase (EC 4.1.1.41)	–	24.441	7	n.d.	–	n.d.	–	n.d.	–
		CoA‐transferase (EC 2.8.3.1)	IPR003702	n.d.	–	n.d.	–	n.d.	–	n.d.	–
Ethanol fermentation		Pyruvate dehydrogenase (EC 1.2.4.1)	IPR017597	n.d.	–	n.d.	–	n.d.	–	n.d.	–
			IPR027110								
		Pyruvate decarboxylase (EC 4.1.1.1)	–	n.d.	–	n.d.	–	n.d.	–	n.d.	–
		Alcohol dehydrogenase (EC 1.1.1.1)	IPR023921	26.691	7	1.563	6	0.337	9	0	0
								0.3203	8		
								0.028	2		
Formic acid fermentation	2,3‐Butanediol fermentation	Pyruvate formate‐lyase (EC 2.3.1.54)	IPR005949	n.d.	–	n.d.	–	n.d.	–	n.d.	–
		Formate Hydrogen Lyase (EC 1.2.1.2)	IPR006478	n.d.	–	n.d.	–	n.d.	–	n.d.	–
			IPR033689								
		Acetolactate synthase (EC 2.2.6.1)	IPR004789	57.686	9	2.784	8	n.d.	–	n.d.	–
			IPR012782	18.144	6	1.922	7				
			IPR012846								
			IPR019455								
		Acetolactate decarboxylase (EC 4.1.1.5)	IPR005128	n.d.	–	n.d.	–	n.d.	–	n.d.	–
		Butanediol dehydrogenase (EC 1.1.1.4)	–	n.d.	–	n.d.	–	n.d.	–	n.d.	–
	Mixed‐acid fermentation	Pyruvate carboxylase (EC 6.4.1.1)	IPR005930	n.d.	–	n.d.	–	n.d.	–	n.d.	–
		Malate dehydrogenase (EC 1.1.1.37)	IPR001252	25.110	7	1.309	5	n.d.	–	0.035	7
			IPR023958								
			IPR011275								
		Fumarase (EC 4.2.1.2)	IPR018951	18.873	6	n.d.	–	0.130	6	n.d.	–
			IPR011167								
		Fumarate reductase (EC 1.3.1.6)	IPR027477	n.d.	–	n.d.	–	n.d.	–	0.007	2
		Lactate dehydrogenase (EC 1.1.1.28)	–	19.016	6	n.d.	–	0.169	7	n.d.	–
				18.631	6			0.189	7		
		Phosphotransacetylase (EC 2.3.1.8)	IPR016475	1.765	2	3.875	8	n.d.	–	0.021	5
			IPR004614								
			IPR002505								
			IPR012147								
		Acetate kinase (EC 2.7.2.1)	IPR000890	79.588	9	4.573	8	0.136	6	0.029	6
			IPR004372								
			IPR023865								
Butyric acid fermentation		Thiolase (EC 2.3.1.9)	–	n.d.	–	n.d.	–	n.d.	–	n.d.	–
		3‐hydroxybutyryl‐ CoA dehydrogenase(EC 1.1.1.157)	–	n.d.	–	n.d.	–	n.d.	–	n.d.	–
		Crotonase (EC 4.2.1.150)	–	n.d.	–	n.d.	–	n.d.	–	n.d.	–
		Butyryl‐CoA dehydrogenase (EC 1.3.8.1)	–	n.d.	–	n.d.	–	n.d.	–	n.d.	–
		Phosphate butyryl transferase (EC 2.3.1.19)	IPR014079	1.765	2	n.d.	–	n.d.	–	n.d.	–
Homoacetogenesis		Butyrate kinase (2.7.2.7) Pyruvate: ferredoxin oxidoreductase (EC 1.2.7.1)	IPR011245	3.494	3	n.d.	–	n.d.	–	0.029	6
			–	50.748	8	14.265	10	0.053	3	0.060	8
				49.909	8					0.050	8
				11.622	5						
				61.543	9						
		Phosphotransacetylase(EC 2.3.1.8)	IPR016475	1.765	2	3.874	8		n.d.	–	0.021
			IPR004614								
			IPR002505								
			IPR012147								
		Acetate kinase (EC 2.7.2.1)	IPR000890	79.588	9	4.573	8	0.136	6	0.029	6
			IPR004372								
			IPR023865								
Lactic acid Fermentation	Homolactic acid fermentation	Glucose‐6‐phosphate isomerase (EC 5.3.1.9)	IPR001672	22.717	6	5.772	9	0.520	9	0.011	3
			IPR010551								
			IPR016758								
			IPR018189								
			IPR023096								
		6‐phospho‐fructokinase (EC 2.7.1.11)	IPR000023	74.683	9	7.921	9	n.d.	–	0.057	8
			IPR012003								
			IPR012004								
			IPR012828								
			IPR015912								
			IPR022953								
		Fructose‐bisphosphate aldolase (EC 4.1.2.13)	IPR023014	152.525	10	15.580	10	1.015	10	n.d.	–
			IPR000741					0.111	5		
			IPR011289								
			IPR029768								
		Triosephosphate isomerase (5.3.1.1)	IPR000652	81.170	9	n.d.	–	n.d.	–	n.d.	–
			IPR020861								
			IPR022891								
			IPR022896								
		Lactate dehydrogenase (EC 1.1.1.28)	–	19.016	6	n.d.	–	0.169	7	n.d.	–
				18.631	6			0.189	7		
	Heterolactic acid fermentation	Hexokinase (EC 2.7.1.1)	IPR001312	81.171	9	n.d.	–	0.115	5	n.d.	–
			IPR019807								
			IPR022672								
			IPR022673								
		Glucose‐6 phosphate dehydrogenase (EC 1.1.1.49)	IPR001282	18.460	6	n.d.	–	0.128	6	n.d.	–
			IPR019796								
			IPR022674								
			IPR022675								
		6‐phosphogluconolactonase (EC 3.1.1.31)	IPR022528	n.d.	–	n.d.	–	0.346	9	n.d.	–
		Phospho‐gluconate dehydrogenase (EC 1.1.1.44)	IPR006184	6.055	3	n.d.	–	0.095	5	n.d.	–
			IPR006114								
			IPR006113								
			IPR006183								
		Ribulose‐phosphate 3‐epimerase (EC 5.1.3.1)	IPR000056	28.915	7	2.740	8	0.578	9	0.012	3
			IPR026019								
		Xylulose‐5‐phosphate phosphoketolase (EC 4.1.2.9)	–	n.d.	–	n.d.	–	n.d.	–	n.d.	–
		Acyl‐phosphatase (EC 3.6.1.7)	IPR001792	15.719	5	n.d.	–	n.d.	–	0.021	5
			IPR017968								
			IPR020456								
			IPR028627								
		Acetate kinase (EC 2.7.2.1)	IPR000890	79.588	9	4.573	8	0.136	6	0.029	6
			IPR004372								
			IPR023865								
		Phosphotransacetylase (EC 2.3.1.8)	IPR016475	1.765	2	3.875	8	n.d.	–	0.028	6
			IPR004614								
			IPR002505								
			IPR012147								
		Acetaldehyde dehydrogenase (EC 1.2.1.10)	IPR003361	n.d.	–	n.d.	–	n.d.	–	n.d.	–
			IPR015426								
		Alcohol dehydrogenase (EC 1.1.1.1)	IPR023921	26.691	7	1.563	6	0.337	9	0	0
								0.303	8		
								0.028	2		

**Table 4:** Proteins possibly associated with syntrophy according to Worm *et al*. (2014) and their respective transcript per million (TPM) values and transcriptional categories for each of the four analysed genome bins. The categories range from 0 (no transcription) and 1 (lowest 10% of transcripts) to 10 (top 10% transcripts). n.d.: not detected

Protein	Subunit	Interpro number	*Thermotogae* bin		*Fusobacteria* bin		*Spirochaetes* bin		*Cloacimonetes* bin	
			TPM	Category	TPM	Category	TPM	Category	TPM	Category
Capsule synthesis protein, CapA	–	IPR019079	n.d.	–	n.d.	–	n.d.	–	0.04	7
Cell cycle, FtsW, RodA SpoVE	–	IPR018365	n.d.	–	0.42	2	n.d.	–	n.d.	–
					0.64	3				
					0.42	2				
Ribonuclease P, conserved site	–	IPR020539	n.d.	–	n.d.	–	n.d.	–	0.12	9
Cytoplasmic FDH	NUO 51 kDa	IPR019575	n.d.	–	n.d.	–	0.054	3	n.d.	–
		IPR001949	79.06	9	0.36	8	0.054	3	0.02	5
									0.02	5
Extracytopl. FDH	Alpha	IPR006443	79.06	9	0.36	8	n.d.	–	0.02	5
FeFe‐hydrogenase	Alpha	IPR004108	n.d.	–	n.d.	–	0.075	4	n.d.	–
		IPR009016	110.75	9	2.07	7	0.075	4	0.05	8
					9.26	10				
		IPR003149	110.75	9	2.07	7	0.075	4	0.05	8
					9.27	10				
		IPR013352	110.75	9	9.27	10	0.075	4	0.05	8
NiFe‐hydrogenase	–	IPR001501	110.75	9	9.27	10	n.d.	–	0.05	8
		IPR018194	n.d.	–	n.d.	–	n.d.	–	n.d.	–
Rnf complex	RnfB	IPR007202	n.d.	–	n.d.	–	n.d.	–	n.d.	–
		IPR010207	798.79	10	2.07	7	0.25	8	0.02	5
					2.68	8				
	RnfC	IPR026902	798.79	10	2.68	8	0.34	9	0.02	5
		IPR010208	14.37	5	4.01	8	0.34	9	0.01	2
	RnfD	IPR004338	14.37	5	4.01	8	3.24	10	0.01	2
		IPR011303	7.63	4	0.93	4	0.39	9	0.01	2
	RnfG	IPR007329	7.63	4	0.93	4	n.d.	–	0.01	2
Ech complex	EchA	IPR001750	1100.12	10	12.01	10	n.d.	–	0.01	2
			162.44	10					0.01	2
		IPR001516	n.d.	–	n.d.	–	n.d.	–	n.d.	–
	EchB	IPR001694	n.d.	–	n.d.	–	n.d.	–	n.d.	–
	EchC	IPR006137	n.d.	–	n.d.	–	n.d.	–	n.d.	–
	EchD	IPR001268	n.d.	–	n.d.	–	n.d.	–	n.d.	–
		IPR012179	n.d.	–	n.d.	–	n.d.	–	n.d.	–
	EchE	IPR001135	n.d.	–	n.d.	–	n.d.	–	n.d.	–
Etf Alpha	–	IPR014731	n.d.	–	n.d.	–	0.41	9	n.d.	–
Etf Beta	–	IPR012255	n.d.	–	n.d.	–	1.19	10	0.03	6
Bcd	–	IPR006089	n.d.	–	n.d.	–	n.d.	–	0.02	5
	–	IPR009075	n.d.	–	n.d.	–	n.d.	–	0.05	8
	–	IPR006092	n.d.	–	n.d.	–	n.d.	–	0.05	8
	–	IPR006091	n.d.	–	n.d.	–	n.d.	–	n.d.	–
	–	IPR013786	n.d.	–	n.d.	–	n.d.	–	0.05	8
	–	IPR009100	n.d.	–	n.d.	–	n.d.	–	0.05	8
DUF224	–	IPR003816	n.d.	–	n.d.	–	n.d.	–	0.05	8
	–	IPR004017	n.d.	–	n.d.	–	n.d.	–	n.d.	–
	–	IPR023234	n.d.	–	n.d.	–	n.d.	–	n.d.	–

### The transcriptional profile of the *Thermotogae* bin indicates a metabolism based on sugar fermentation

**Figure 1:**
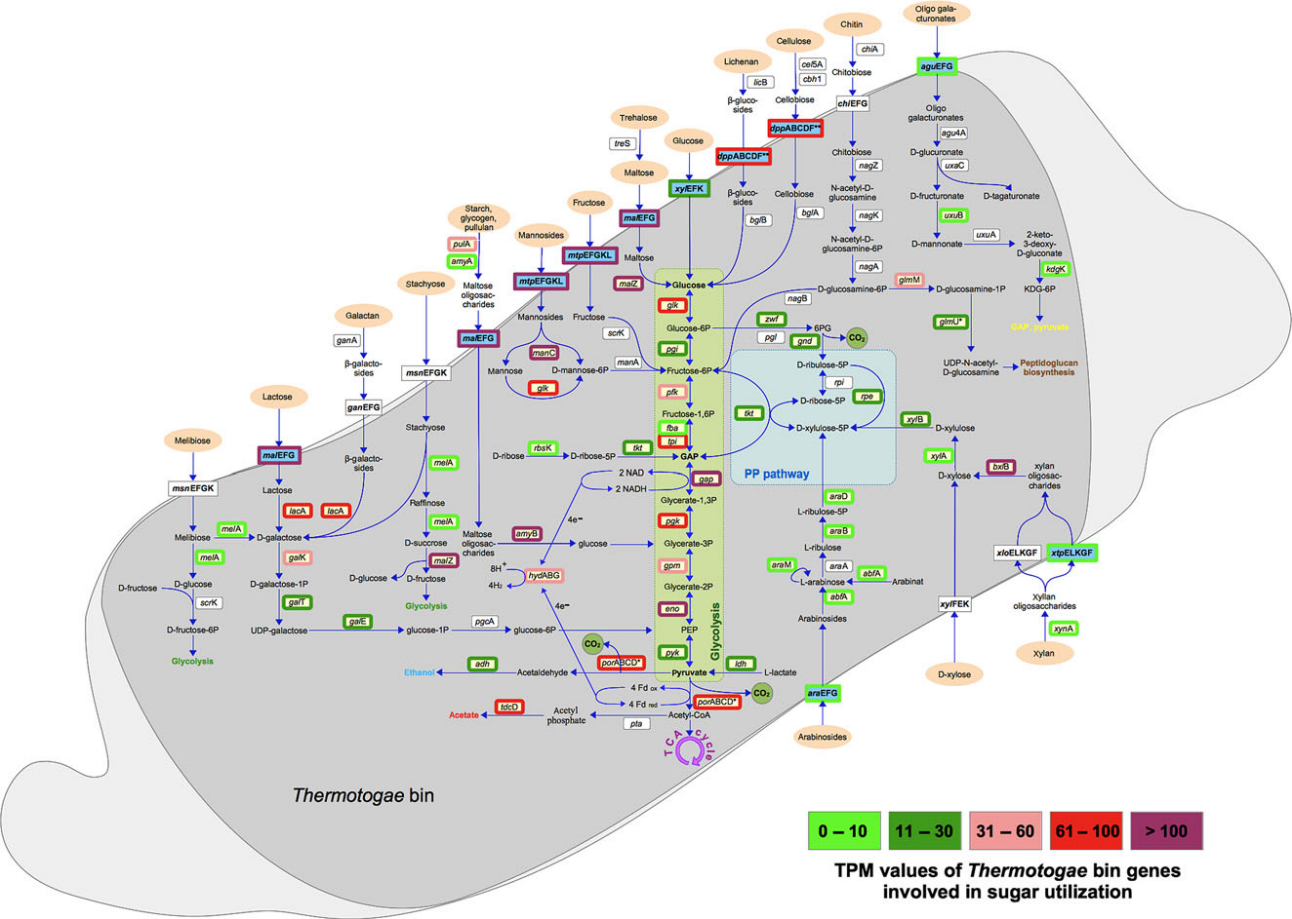
Metabolic reconstruction of sugar utilization pathways in the *Thermotogae* genome bin and transcript per million (TPM) values for genes encoding involved proteins. Figure modified according to Maus *et al*. (2016). Carbohydrates are labelled in light orange ovals, transporters in blue and corresponding genes in yellow rectangles. White rectangles represent genes lacking in the genome bin in comparison with its reference strain *Defluviitoga tunisiensis* L3. Frames indicate the category of TPM values for the respective gene, the five categories explained on the bottom right. The glycolysis and the pentose phosphate pathways are highlighted in green and blue, respectively. Abbreviations: CO_2_, carbon dioxide; GAP, glyceraldehyde‐3‐phosphate; KDG‐6P, 2‐keto‐3‐deoxy‐d‐gluconase‐6‐phosphate.

The previous taxonomic and genetic analyses of the *Thermotogae* genome bin showed that it represents a species closely related to the thermophilic bacterium *Defluviitoga tunisiensis* L3 (Maus *et al*., 2015, 2016), or may even indicate another strain of this species. It is presumably able to utilize a wide variety of mono‐, di‐ and polysaccharides and was predicted to produce acetate, hydrogen and carbon dioxide as end‐products (Stolze *et al*., 2016). Analysis of the 25 most highly transcribed genes within the *Thermotogae* bin showed that 22 of them are functionally classified and 17 encode proteins involved in mandatory processes like transcription, translation, fatty acid metabolism, iron storage, electron transport, protein and RNA folding. Three highly transcribed genes encode proteins associated with ATP‐binding cassette (ABC) transporters (see Table S1), known as importers for sugars and other solutes (Davidson *et al*., 2008), with two of them specifically annotated as maltose import system. In total, 223 transcripts encoding proteins involved in sugar utilization were found for the bin. Most of these are involved in either binding and import (ABC sugar transporters) or sugar utilization within the cell, e.g. *via* the glycolysis pathway. Table 2 shows that the *Thermotogae* bin encodes eleven glycoside hydrolase (GH) family proteins, all of them being transcribed featuring TPM values above the average (TPM categories ≥ 6). Selected sugar utilization genes of the genome bin and their transcriptional activity are indicated in Fig. 1. It appeared that especially the sugar transporter genes and those for glycolysis enzymes are highly transcribed. In general, these findings strongly suggest that this species actively degrades and utilizes a variety of carbohydrates, whose end‐products are further channelled into the glycolysis pathway.

Regarding the production of end‐products, Fig. 1 and Table 3 reflect the bin's activity in hydrogen, CO_2_, acetate and possibly lactate and ethanol production. Alternatively, lactate could be used for pyruvate production. Hydrogen and acetate production is known for *D. tunisiensis* L3 and was previously discussed as hint for a syntrophic lifestyle with aceticlastic or hydrogenotrophic *Archaea* (Maus *et al*., 2016; Stolze *et al*., 2016). This assumption is further supported by the presence and partially high transcriptional activities of genes encoding enzymes associated with syntrophy (Worm *et al*., 2014). The same applies for genes that are important for hydrogen production (see Table 4 and Fig. 1). For example, transcripts encoding the bifurcating FeFe‐hydrogenase (*hydABG*), pyruvate oxidoreductase (*porABCD*) and subunits of the Rnf complex were identified for the genome bin. The Rnf complex represents a membrane bound transporter that was shown to be able to conserve energy by coupling the oxidation of NADH to the simultaneous reduction of ferredoxin with ion transport (Biegel and Muller, 2010; Hess *et al*., 2013; Worm *et al*., 2014). The reduced ferredoxin may then be used as electron donor by a (bifurcating) hydrogenase to form H_2_ from protons (H^+^). The consumption of hydrogen from the producer would enable its formation despite the thermodynamically unfavourable nature of this process (Verhaart *et al*., 2010; Worm *et al*., 2014). Despite the lack of other putative syntrophy‐associated genes, the high TPM values for hydrogen production‐associated genes strongly indicate that the species represented by the *Thermotogae* bin is syntrophically associated with a partner consuming hydrogen, as it was previously described for other *Thermotogae* species (Balk *et al*., 2002; Johnson *et al*., 2006).

In summary, the *in situ* transcriptional profile of the *Thermotogae* genome bin shows partially high transcriptional activities regarding genes encoding proteins involved in (complex) sugar utilization, acetate, ethanol, CO_2_ and H_2_ production and those having predicted functions in a syntrophic association. The profile therefore reflects a possibly syntrophic and sugar‐based lifestyle of the corresponding species that in its thermophilic habitat occupies the role of a hydrolytic/acetogenic bacterium.

### The transcriptional profile of the *Fusobacteria* bin indicates a motile species with a metabolism based on amino acid fermentation

Previous genetic analyses of the *Fusobacteria* genome bin suggested that the corresponding bacterium is an amino acid‐fermenting, acetogenic bacterium, possibly also syntrophically associated with methanogenic *Archaea* (Stolze *et al*., 2016). The species’ transcriptome analysis, based on metatranscriptome sequencing data from its mesophilic habitat, was supposed to uncover its *in situ* response to prevailing environmental conditions. Analyses of the genes with the 25 highest TPM values showed functional annotations for 15 genes, with six being involved in mandatory processes of translation, chromosome and RNA protection, reactive oxygen species (ROS) scavenging, fatty acid metabolism and cell division (see Table S2). Interestingly, eight transcripts encode flagellum‐associated proteins. In total, the bin encodes 46 proteins of this functional context and additional 73 proteins involved in chemotaxis (e.g. histidine kinases, Che proteins) (Bi and Lai, 2015; Micali and Endres, 2016), most of them being highly transcribed (category 8). Regarding the bin's metabolism, the top 25 list of the most highly transcribed genes did not provide any information, but Table 2 shows that the *Fusobacteria* bin features only three encoded GH families, however with transcriptional categories of 3, 6 and 10. Still, the low number of transcribed GH family genes and their predicted functional context rather suggest that the species represented by the bin does not utilize (complex) sugars. Previous analyses on the genetic content suggested that its metabolism is based on glutamate and lysine fermentation (Stolze *et al*., 2016). Transcriptional analyses showed that for glutamate utilization, some of the key enzymes of the hydroxyglutarate pathway have high (category 8), some others lower TPM values (categories 2 and 3). For lysine utilization, the pathway of d‐ and l‐lysine degradation to acetate is complete, with medium TPM values (category 6); both findings strongly indicate an active amino acid utilization.

The transcriptional data also clearly indicate ethanol, acetate, H_2_, CO_2_ and possibly lactate as end‐products of the species’ metabolism, as shown in Table 3. The key genes encoding alcohol dehydrogenase, lactate dehydrogenase and acetate kinase feature transcription categories of 6, 8 and 8 respectively. Table 3 also shows that hydrogen production is likely to occur as the pyruvate:ferredoxin oxidoreductase, converting pyruvate to acetyl‐CoA and CO_2_ and simultaneously reducing ferredoxin, is highly transcribed (categories 5, 8 and 9). Ferredoxin is, i.a., used for hydrogen production (Biegel and Muller, 2010; Hess *et al*., 2013) and features transcriptional categories of 10 (see Table S2). It can be used by a bifurcating FeFe‐hydrogenase to produce hydrogen. The genes encoding this enzyme are highly transcribed (categories 7 and 10) as shown in Table 4, which summarizes transcriptional activities of genes encoding proteins involved in syntrophy according to Worm *et al*. (2014). As the described reaction is thermodynamically unfavourable, it depends on a hydrogen‐consuming methanogenic archaeon (Sieber *et al*., 2012). The hypothesis of a possible syntrophic association is supported by other transcripts encoding putative syntrophy‐associated genes being highly transcribed by the species, and among them, subunits of the Rnf complex with transcriptional categories between 4 and 8 (Table 4).

However, these findings are not in line with the findings indicating a motile lifestyle, as it is known that the formation of mats or biofilms eventually results in motility loss (Alexandre, 2015). Flagella play important roles in the maintenance of the close physical contact between the syntrophic partners (McInerney *et al*., 2009; Krumholz *et al*., 2015), but this does not explain the high transcription rates of chemotaxis‐associated genes. However, the metatranscriptome‐based profile comprises the whole *Fusobacteria* bin*‐*represented population *in situ*. A subpopulation may be syntrophically associated, while other cells still were motile.

In summary, the transcriptional profile of the *Fusobacteria* genome bin, as deduced from metatranscriptome sequencing data, depicts a motile, acidogenic, mostly amino acid‐based metabolism with acetate, ethanol, CO_2_, H_2_ and probably lactate as fermentation end‐products.

### The transcriptional profile of the *Spirochaetes* bin indicates a sugar fermentation‐based species

Previously, the *Spirochaetes* genome bin was analysed genetically and as deduced from its metabolic potential, the bin may constitute a syntrophic sugar‐fermenting bacterium producing acetate, CO_2_ and H_2_ (Stolze *et al*., 2016). Based on metatranscriptome sequencing data from the mesophilic BGP, the bin's activity and role within its habitat were analysed at the transcriptional level. Regarding the top 25 list of the most highly transcribed genes, 19 encoded gene products received functional annotations, with 15 of them being involved in mandatory processes of translation, transcription, fatty acid metabolism, protein folding and export and electron transfer (Table S3). Two of the annotated genes among the 25 most highly transcribed ones encode an ABC transporter substrate‐binding protein and a LacI family transcriptional regulator, both featuring transcriptional categories 10. ATP‐binding cassette (ABC) transporters are known as importers for sugars and also other solutes, while LacI family proteins function as transcription inhibitors for genes encoding proteins for lactose utilization (Davidson *et al*., 2008; Santillan and Mackey, 2008; Camas *et al*., 2010). In total, 290 genes (14% of all genes) encoding proteins involved in sugar import and utilization are present, all but six of them being transcribed. 48 genes encode (ABC) transporters directly associated with sugar import, mostly unspecific, some specific for lactose and the monosaccharides arabinose, rhamnose, ribose, fructose and xylose (categories 2 – 9). Additionally, Table 2 shows that the bin actively transcribed genes representing seven glycoside hydrolase families with transcriptional categories between 2 and 6.

Regarding the species’ fermentation end‐products, the transcriptional data strongly indicate the release of CO_2_, H_2_, acetate, ethanol and probably lactate. As shown in Table 3, the key genes for the production of the latter three compounds show high transcriptional categories. Additionally, high transcriptional categories of the encoded ferredoxin‐reducing and CO_2_‐producing pyruvate: ferredoxin oxidoreductase (see Table 3), ferredoxin (categories 4 and 9) and a bifurcating FeFe‐hydrogenase (category 10, Table 4) indicate hydrogen production from NADH and ferredoxin.

According to Worm *et al*. (2014), the bifurcating FeFe‐hydrogenase belongs to those enzymes possibly involved in syntrophy, as summarized in Table 4. Some other genes within this Table are actively transcribed by the analysed species, and among them, the majority of the Rnf complex subunit genes. They feature transcriptional categories between 8 and 10 and therefore belong to the most highly transcribed genes of this organism. It was proposed that the Rnf complex may play a crucial role in syntrophy (Worm *et al*., 2014), and its high transcription rate indicates a possible syntrophic interaction of the studied species at the time of RNA extraction. However, as only a small number of putative syntrophy‐associated genes are transcribed, this conclusion cannot be drawn with certainty, but in the context of hydrogen production and the seemingly acetogenic lifestyle, it is a likely assumption.

Summarizing, the metatranscriptome data mapping onto the *Spirochaetes* bin enabled *in situ* transcriptomic profiling of this so far unknown and uncharacterized species. Its metabolism seems to be mainly based on monosaccharides, but may also involve the degradation of some complex di‐ and polysaccharides. Transcriptional activities for associated genes also indicated acetate, ethanol, possibly lactate, CO_2_ and H_2_ as fermentation end‐products. A syntrophic association with methanogenic *Archaea* is presumed, due to the transcription of genes encoding proteins associated with syntrophy and hydrogen production, especially a bifurcating hydrogenase.

### The transcriptional profile of the *Cloacimonetes* bin indicates an amino acid fermentation‐based species

In a previous study, the genome bin assigned to the phylum *Cloacimonetes* was analysed on the genetic level, concluding that it probably represents an amino acid‐fermenting, CO_2_ and H_2_‐producing bacterium, possibly syntrophically associated with methanogenic *Archaea* (Stolze *et al*., 2016). Mapping of metatranscriptome sequencing data from the mesophilic BGP on the *Cloacimonetes* genome bin was supposed to give insights into the *in situ* transcriptomic activity of the corresponding species and enable the uncovering of its response to prevailing environmental conditions.

Analyses of the 25 most highly transcribed genes, according to their TPM values, showed that 16 genes could be identified to encode proteins involved in mandatory bacterial functions like translation, chromosome structure maintenance, fatty acid metabolism and protein transport and protection (see Table S4). Interestingly, there are no other transcripts among them that encode proteins showing a certain response to the species’ environment or being involved in the genome bins’ postulated metabolism based on amino acids.

However, Table 2 shows that five encoded glycoside hydrolases feature only low transcription categories except for those two predicted to be involved in cell wall degradation. Generally, the bin lacks genes encoding proteins for the utilization of sugars; the only exception is glucose degradation *via* the glycolysis pathway whose enzymes are completely encoded and feature transcriptional categories between 2 and 9. However, the transcriptional data clearly show that fermentation of the amino acids glutamate, lysine, alanine, asparagine, aspartate, cysteine and proline is preferred by the bacterium. Genes encoding proteins involved in their conversion into pyruvate were identified featuring transcriptional activity categories between 5 and 9.

The end‐product of these pathways is most likely acetate, as all enzymes for its production are encoded and transcribed with categories between 5 and 8, while the other fermentation pathways are largely incomplete (Table 3). Interestingly, the studied species may have the potential to produce ethanol; however, no transcripts for the key enzyme, the alcohol dehydrogenase, were identified. This indicates that the *Cloacimonetes* species represents an acetogenic bacterium.

However, other end‐products seem to be CO_2_ and H_2_. Carbon dioxide is produced mainly *via* the conversion of pyruvate to acetyl‐CoA by the pyruvate:ferredoxin oxidoreductase (see Table 3), simultaneously reducing ferredoxin in the process. In addition to this, genes encoding the bifurcating FeFe‐hydrogenase using NADH and ferredoxin to produce hydrogen (Sieber *et al*., 2012) are actively transcribed and show high TPM values (see Table 4). Also, a second Fe‐only hydrogenase (transcriptional categories 8 to 10) and its assembly protein (categories 3–6) are transcribed by the *Cloacimonetes* genome bin. Transcripts encoding ferredoxins were also found, with categories 5 and 10 (not shown). These findings strongly indicate the production of hydrogen *via* this thermodynamically unfavourable reaction. Additionally, it indicates that this species is likely to be syntrophically associated with hydrogenotrophic (or aceticlastic) *Archaea*. This is also supported by partially high transcriptional activities of genes encoding proteins presumably associated with syntrophy (Table 4).

In summary, the metatranscriptome‐based profile of the *Cloacimonetes* bin showed that it actively ferments amino acids, producing acetate, H_2_ and CO_2_ in the process and is very likely associated with hydrogenotrophic and aceticlastic *Archaea*. The used methods of metatranscriptome sequencing and genome bin‐enabled transcriptional profiling therefore proved to be valuable tools for *in situ* characterization of unknown species and to deduce their role and importance within biogas‐producing communities.

## Experimental procedures

### Total microbial RNA extraction from three mesophilic and one thermophilic production‐scale biogas plants

Fermentation samples for whole‐microbial community RNA extraction were taken from three mesophilic and one thermophilic production‐scale biogas plants in Germany as described in Stolze *et al*. (2016). RNA extraction is based on acid phenol treatment followed by usage of the RNeasy Midi bacteria Kit (Qiagen, Hilden, Germany) and DNA digestion using DNase by Roche (Mannheim, Germany) and Qiagen. Whole‐community RNA was prepared from fermenter samples applying the protocol as follows: 4.4 g of fermenter sludge and 2.5 ml TE buffer (4°C) were mixed and applied on a nylon filter (40 μm nylon BD Biosciences, Heidelberg, Germany). Centrifugation at 400 *g* and 4°C for 2 min and filtrate mixing 1:1 (v/v) with acid phenol (4°C) followed. The mixture was added to 0.5 g glass beads (0.1 mm) in a 15 ml tube, vortexed for 4 min at the highest level, followed by centrifugation at 5000 *g* for 5 min. The upper phase was then mixed 1:4 (v/v) with RLT buffer (RNeasy Midi Kit, with 2‐Mercaptoethanol). Next steps followed the RNeasy Midi bacteria protocol (Qiagen) starting at step 5, with the upper phase/ethanol ratio being 1:2.8 (v/v) (without RLT). Finally, RNA was eluted in 150 μl RNase‐free water and remaining DNA was removed using DNase I by Roche and the RNeasy Mini kit by Qiagen following the manufacturer's instructions. Quality and quantity of the extracted RNA were evaluated using a Prokaryote RNA 600 Pico chip and the Agilent 2100 Bioanalyzer (Agilent, Santa Clara, CA, USA).

For the mesophilic BGPs 1, 2 and 3, and the thermophilic BGP4, RNA extraction was performed in duplicates. cDNA library preparation for metatranscriptome sequencing and metagenome/metatranscriptome sequencing was performed at the DOE Joint Genome Institute (JGI) in Walnut Creek, CA, USA.

### rRNA depletion, library preparation and high‐throughput metatranscriptome sequencing

For all eight whole RNA samples, rRNA depletion, cDNA library preparation and metatranscriptome sequencing were performed at the DOE JGI. Library preparation was performed following the TruSeq Stranded Total RNA sample preparation guide by Illumina (San Diego, CA, USA). Prior to library generation, rRNA depletion was performed using the Ribo‐Zero rRNA Removal Kit (Bacteria) (Epicentre, Chicago, IL, USA). For library construction, the TruSeq Stranded mRNA Sample Preparation Kit (Illumina; San Diego, CA, USA) was used. Depleted mRNA was fragmented and reverse‐transcribed using the Superscript II reverse transcriptase (Invitrogen, Waltham, MA, USA). After second‐strand synthesis, end‐repair, A‐tailing, adapter ligation of the double‐stranded cDNA and 10 cycles of PCR amplification followed.

Quantification of metatranscriptome libraries was performed using the next‐generation sequencing library qPCR kit (Kapa Biosystems, Wilmington, DE, USA) and the LightCycler 480 real‐time PCR instrument (Roche, Basel, Switzerland). Sequencing preparation was performed using the TruSeq paired‐end cluster kit (v3, Illumina; San Diego, CA, USA). Finally, metatranscriptome sequencing was performed on the HiSeq 2000 sequencer using the TruSeq SBS sequencing kits (v3) following the 2 × 150 indexed high‐output run instruction (both by Illumina).

### Metatranscriptome sequence data processing

In order to determine the transcriptional profiles of the four genome bins, metatranscriptome data of the BGPs they derived from were used: BGP3 (*Fusobacteria*,* Spirochaetes* and *Cloacimonetes* (WWE1) bin), hereinafter referred to as the mesophilic BGP, and BGP4 (*Thermotogae* bin), hereinafter referred to as the thermophilic BGP. In total, 9001.8 million reads (137 269 Gbp; Table 1) were generated, the deepest sequencing of biogas metatranscriptomes so far. Kallisto (Bray *et al*., 2016; version 0.42.5) was used to quantify abundances of transcripts. The coding sequences predicted from contigs of the combined assembly of the four metagenome samples (see Stolze *et al*., 2016) were used as input to build the transcriptome index using kallisto index. FASTQ files from each metatranscriptome sample were ‘pseudoaligned’ using kallisto quant with default parameters. Transcripts per million (TPM) values for each gene were extracted from the resulting abundance file, for which the number of reads mapping on a gene is normalized on gene length and data set size.

Annotation of predicted genes of the metagenome assembly (see Stolze *et al*., 2016) was performed using InterProScan version 5.24‐63.0.

To predict genes encoding carbohydrate‐active enzymes, the carbohydrate‐active enzyme database (CAZy) annotation web server dbCAN (Yin *et al*., 2012) was used.

## Conflict of interest

None declared.

## Data sets

The datasets supporting the conclusions of this article are available in the short read archive (SRA, Metatranscriptome sequencing data): Metatranscriptome Data mesophilic BGP: https://www.ncbi.nlm.nih.gov/sra/?term=SRP096994, https://www.ncbi.nlm.nih.gov/sra/?term=SRP096993. Metatranscriptome Data thermophilic BGP: https://www.ncbi.nlm.nih.gov/sra/?term=SRP096995, https://www.ncbi.nlm.nih.gov/sra/?term=SRP096996.

## Supporting information


**Table S1.** The 25 most highly transcribed genes of the *Thermotogae* bin, by Transcripts Per Million (TPM) values, their encoded proteins and functional contexts.

**Table S2.** The 25 most highly transcribed genes of the *Fusobacteria* bin, by Transcripts Per Million (TPM) values, their encoded proteins and functional contexts.

**Table S3.** The 25 most highly transcribed genes of the *Spirochaetes* bin, by Transcripts Per Million (TPM) values, their encoded proteins and functional contexts.

**Table S4.** The 25 most highly transcribed genes of the *Cloacimonetes* bin, by Transcripts Per Million (TPM) values, their encoded proteins and functional contexts.

**Table S5.** Unshortened table of glycosyl hydrolase (GH) families, their respective Transcript per Million (TPM) values and transcription categories between 0 and 10 for all four genome bins.

**Table S6.** Unshortened table of fermentation pathway proteins, their respective Transcript per Million (TPM) values and transcription categories between 0 and 10 for all four genome bins.

**Table S7.** Unshortened table of possibly syntrophy associated proteins, their respective Transcript per Million (TPM) values and transcription categories between 0 and 10 for all four genome bins.
