# Supplementary material for: Targeted in situ metatranscriptomics for selected taxa from mesophilic and thermophilic biogas plants
Source: Microb Biotechnol. 2017 Dec 4;11(4):667–79. doi: 10.1111/1751-7915.12982 (PMC6011919; doi:10.1111/1751-7915.12982)
Supplement: Supplementary file 1 — Table S1. The 25 most highly transcribed genes of the Thermotogae bin, by Transcripts Per Million (TPM) values, their encoded proteins and functional contexts. [file MBT2-11-667-s001.docx]

**Supplementary table 1:** The 25 most highly transcribed genes of the *Thermotogae* bin, as determined by Transcripts Per Million (TPM) values, their encoded proteins and functional contexts.

| **Position (out of 1,918)** | **TPM in thermophilic BGP** | **Encoded Protein** | **Functional context** |
| --- | --- | --- | --- |
| 1 | 20071.8 | Hypothetical protein | - |
| 2 | 14277.3 | Cold-shock protein | RNA folding and protection |
| 3 | 5041.24 | Histone-like DNA-binding protein | DNA packing / regulation |
| 4 | 4980.2 | RNA-binding protein | Transcription |
| 5 | 4972.0 | DNA-directed RNA polymerase, Ω subunit | Transcription |
| 6 | 2933.2 | Hypothetical protein | - |
| 7 | 2653.7 | 50S ribosomal protein L31 | Translation |
| 8 | 1894.4 | Stage V sporulation protein S | Unknown |
| 9 | 1880.8 | Heat shock protein Hsp20 | Protein folding |
| 10 | 1787.8 | 50S ribosomal protein L28 | Translation |
| 11 | 1665.8 | Ferredoxin | (assists in) H_2_ production |
| 12 | 1661.5 | ABC transporter (trehalose/maltose specific) | Sugar import |
| 13 | 1648.5 | Thymidylate synthase | DNA replication |
| 14 | 1478.3 | 50S ribosomal protein L32 | Translation |
| 15 | 1319.5 | ABC transporter substrate-binding protein | Solute import |
| 16 | 1219.6 | Hypothetical protein | - |
| 17 | 1125.7 | 50S ribosomal protein L35 | Translation |
| 18 | 1100.1 | FMN-binding domain-containing protein | Redox process/ electron-transfer |
| 19 | 1040.5 | Maltose-transporting ATPase | Sugar import |
| 20 | 891.5 | Transcriptional regulator | Transcription |
| 21 | 827.7 | RNA polymerase sigma factor rpoD | Transcription |
| 22 | 798.7 | Putative NADH:ubiquinone oxidoreductase, subunit RnfB | Ion transport |
