# Supplementary material for: Targeted in situ metatranscriptomics for selected taxa from mesophilic and thermophilic biogas plants
Source: Microb Biotechnol. 2017 Dec 4;11(4):667–79. doi: 10.1111/1751-7915.12982 (PMC6011919; doi:10.1111/1751-7915.12982)
Supplement: Supplementary file 2 — Table S2. The 25 most highly transcribed genes of the Fusobacteria bin, by Transcripts Per Million (TPM) values, their encoded proteins and functional contexts. [file MBT2-11-667-s002.docx]

**Supplementary table 2:** The 25 most highly transcribed genes of the *Fusobacteria* bin, as determined by Transcripts Per Million (TPM) values, their encoded proteins and functional contexts.

| **Position (out of 1931)** | **TPM in mesophilic BGP** | **Encoded Protein** | **Functional context** |
| --- | --- | --- | --- |
| 1 | 1466.1 | Hypothetical protein | - |
| 2 | 318.4 | Flagellin | Motility |
| 3 | 234.0 | Hypothetical protein | - |
| 4 | 201.3 | Cold-shock protein | RNA folding and protection |
| 5 | 196.7 | Flagellin | Motility |
| 6 | 177.3 | Hypothetical protein | - |
| 7 | 147.2 | Flagellin | Motility |
| 8 | 145.6 | Flagellar biosynthesis anti-sigma factor FlgM | Motility |
| 9 | 122.5 | Flagellin | Motility |
| 10 | 111.4 | Flagellin | Motility |
| 11 | 109.4 | DNA-binding protein | Chromosomal protein |
| 12 | 85.2 | Hypothetical protein | - |
| 13 | 79.8 | Membrane protein | - |
| 14 | 72.27 | Hypothetical protein | - |
| 15 | 66.8 | Hypothetical protein | - |
| 16 | 65.3 | Septation protein spoVG | Sporulation stage V |
| 17 | 63.4 | Ferredoxin | (assists in) H_2_ production |
| 18 | 61.3 | Hypothetical protein | - |
| 19 | 59.5 | Flagellin | Motility |
| 20 | 57.7 | Flagellin | Motility |
| 21 | 52.7 | Acyl carrier protein | Fatty acid synthesis |
| 22 | 52.6 | Hypothetical protein | - |
| 23 | 43.8 | Rubredoxin | Electron-transfer/ROS scavenging |
| 24 | 43.6 | 30S ribosomal protein | Translation |
| 25 | 43.5 | Hypothetical protein | - |
