# Supplementary material for: Targeted in situ metatranscriptomics for selected taxa from mesophilic and thermophilic biogas plants
Source: Microb Biotechnol. 2017 Dec 4;11(4):667–79. doi: 10.1111/1751-7915.12982 (PMC6011919; doi:10.1111/1751-7915.12982)
Supplement: Supplementary file 3 — Table S3. The 25 most highly transcribed genes of the Spirochaetes bin, by Transcripts Per Million (TPM) values, their encoded proteins and functional contexts. [file MBT2-11-667-s003.docx]

**Supplementary table 3:** The 25 most highly transcribed genes of the *Spirochaetes* bin, as determined by Transcripts Per Million (TPM) values, their encoded proteins and functional contexts.

| **Position (out of 2094)** | **TPM in mesophilic BGP** | **Encoded Protein** | **Functional context** |
| --- | --- | --- | --- |
| 1 | 22.8 | Heat shock protein Hsp20 | Protein folding |
| 2 | 20.2 | Heat shock protein Hsp20 | Protein folding |
| 3 | 18.5 | Hypothetical protein | - |
| 4 | 11.8 | 50S ribosomal protein L34 | Translation |
| 5 | 11.8 | 50S ribosomal protein L34 | Translation |
| 6 | 9.2 | 50S ribosomal protein L31 | Translation |
| 7 | 8.8 | RNA-binding protein | Transcription |
| 8 | 8.8 | Hypothetical protein | - |
| 9 | 8.6 | RNA polymerase subunit sigma-24 | Heat shock response |
| 10 | 8.6 | 50S ribosomal protein L33 | Translation |
| 11 | 8.0 | Rubredoxin | Redox process/electron-transfer |
| 12 | 7.1 | Hypothetical protein | - |
| 13 | 6.3 | 50S ribosomal protein L21 | Translation |
| 14 | 5.6 | 30S ribosomal protein S19 | Translation |
| 15 | 5.6 | 50S ribosomal protein L27 | Translation |
| 16 | 5.5 | 50S ribosomal protein L32 | Translation |
| 17 | 5.5 | Preprotein translocase subunit SecE | Protein export |
| 18 | 5.3 | Co-chaperonin GroES | Protein folding |
| 19 | 5.1 | Hypothetical protein | - |
| 20 | 5.0 | Acyl carrier protein | Fatty acid synthesis |
| 21 | 4.9 | RNA-binding protein | Transcription |
| 22 | 4.9 | ABC transporter substrate-binding protein | Solute import |
| 23 | 4.9 | LacI family transcriptional regulator | Regulation of lactose metabolism |
| 24 | 4.8 | Hypothetical protein | - |
| 25 | 4.6 | Hypothetical protein | - |
