# Supplementary material for: Targeted in situ metatranscriptomics for selected taxa from mesophilic and thermophilic biogas plants
Source: Microb Biotechnol. 2017 Dec 4;11(4):667–79. doi: 10.1111/1751-7915.12982 (PMC6011919; doi:10.1111/1751-7915.12982)
Supplement: Supplementary file 4 — Table S4. The 25 most highly transcribed genes of the Cloacimonetes bin, by Transcripts Per Million (TPM) values, their encoded proteins and functional contexts. [file MBT2-11-667-s004.docx]

**Supplementary table 4:** The 25 most highly transcribed genes of the *Cloacimonetes* bin, as determined by Transcripts per Million (TPM) values, their encoded proteins and functional contexts.

| **Position (out of 1473)** | **TPM in mesophilic BGP** | **Encoded Protein** | **Functional context** |
| --- | --- | --- | --- |
| 1 | 129.3 | Hypothetical protein | - |
| 2 | 23.2 | Hypothetical protein | - |
| 3 | 20.7 | 50S ribosomal protein L32 | Translation |
| 4 | 9.0 | Integration host factor subunit beta | Chromosomal protein |
| 5 | 8.7 | Hypothetical protein | - |
| 6 | 7.0 | Hypothetical protein | - |
| 7 | 6.8 | Hypothetical protein | - |
| 8 | 5.8 | Hypothetical protein | - |
| 9 | 5.2 | 50S ribosomal protein L36 | Translation |
| 10 | 4.2 | Hypothetic protein | - |
| 11 | 2.6 | 50S ribosomal protein L33 | Translation |
| 12 | 1.8 | Putative signal peptide | Protein export (putative) |
| 13 | 1.7 | DNA-binding protein | Chromosomal protein |
| 14 | 1.6 | Translation initiation factor IF-3 | Translation |
| 15 | 1.5 | DNA-binding protein | Chromosomal protein |
| 16 | 1.4 | 30S ribosomal protein S8 | Translation |
| 17 | 1.2 | Acyl carrier protein | Fatty acid synthesis |
| 18 | 1.1 | Signal peptide (putative) | Protein export (putative) |
| 19 | 1.0 | 30S ribosomal protein S15 | Translation |
| 20 | 1.0 | 50S ribosomal protein L28 | Translation |
| 21 | 0.9 | Isoleucyl-tRNA synthase | Translation |
| 22 | 0.9 | Phosphate acyltransferase PlsX | Phospholipid metabolism |
| 23 | 0.9 | Heat shock protein Hsp20 | Protein folding |
| 24 | 0.8 | 50S ribosomal protein L11 | Translation |
| 25 | 0.8 | Hypothetical protein | - |
