# Supplementary material for: Targeted in situ metatranscriptomics for selected taxa from mesophilic and thermophilic biogas plants
Source: Microb Biotechnol. 2017 Dec 4;11(4):667–79. doi: 10.1111/1751-7915.12982 (PMC6011919; doi:10.1111/1751-7915.12982)
Supplement: Supplementary file 5 — Table S5. Unshortened table of glycosyl hydrolase (GH) families, their respective Transcript per Million (TPM) values and transcription categories between 0 and 10 for all four genome bins. [file MBT2-11-667-s005.docx]

**Supplementary Table 5:** Unshortened table of glycosyl hydrolase (GH) families, their respective Transcript per Million (TPM) values and transcription categories between 0 and 10 for all four genome bins.

| **Enzymes** | | ***Thermotogae* bin** | | ***Fusobacteria* bin** | | ***Spirochaetes* bin** | | ***Cloacimonetes* bin** | |
| --- | --- | --- | --- | --- | --- | --- | --- | --- | --- |
| **Enzyme type** | **Glycoside Hydrolase Family** | **TPM** | **Category** | **TPM** | **Category** | **TPM** | **Category** | **TPM** | **Category** |
| **Endo - and exo-1,4-β-D-glucanase (cellulase)** | **GH5** | 3.639 | 3 | n.d. | - | n.d. | - | n.d. | - |
|  | **GH8** | n.d. | - | n.d. | - | n.d. | - | n.d. | - |
|  | **GH9** | n.d. | - | n.d. | - | n.d. | - | n.d. | - |
|  | **GH44** | n.d. | - | n.d. | - | n.d. | - | n.d. | - |
|  | **GH48** | n.d. | - | n.d. | - | n.d. | - | n.d. | - |
|  | **GH124** | n.d. | - | n.d. | - | n.d. | - | n.d. | - |
| **Hemicellulose** | **GH10** | n.d. | - | n.d. | - | n.d. | - | n.d. | - |
|  | **GH11** | n.d. | - | n.d. | - | n.d. | - | n.d. | - |
|  | **GH16** | 4.340 | 3 | n.d. | - | n.d. | - | 0 | 0 |
|  | **GH28** | 2.167 | 2 | n.d. | - | 0.023 | 2 | n.d. | - |
|  | **GH30** | n.d. | - | n.d. | - | n.d. | - | n.d. | - |
|  | **GH53** | 41.236 | 8 | n.d. | - | n.d. | - | n.d. | - |
|  | **GH74** | n.d. | - | n.d. | - | n.d. | - | n.d. | - |
|  | **GH81** | n.d. | - | n.d. | - | n.d. | - | n.d. | - |
|  | **GH113** | n.d. | - | n.d. | - | n.d. | - | n.d. | - |
|  | **GH115** | n.d. | - | n.d. | - | 0.132 | 6 | n.d. | - |
|  | **GH76** | n.d. | - | n.d. | - | 0.018 | 1 | n.d. | - |
| **Starch and glycogen hydrolase** | **GH13** | 3.958 | 3 | 1.574 | 6 | 0.129 | 6 | 0.007 | 2 |
|  | **GH15** | n.d. | - | n.d. | - | n.d. | - | n.d. | - |
|  | **GH27** | n.d. | - | n.d. | - | n.d. | - | n.d. | - |
|  | **GH77** | n.d. | - | 9.716 | 10 | 0.102 | 6 | 0 | - |
|  | **GH57** | 35.201 | 8 | 3.788 | 8 | 0.165 | 7 | 0.021 | 5 |
|  | **GH126** | n.d. | - | n.d. | - | n.d. | - | n.d. | - |
| **Lysozyme, chitinase (cell wall degradation)** | **GH18** | 6.172 | 3 | n.d. | - | n.d. | - | 0.021 | 5 |
|  | **GH23** | 20.029 | 6 | 0.655 | 3 | n.d. | - | 0.063 | 8 |
|  | **GH24** | n.d. | - | n.d. | - | n.d. | - | n.d. | - |
|  | **GH25** | n.d. | - | n.d. | - | n.d. | - | n.d. | - |
|  | **GH73** | n.d. | - | n.d. | - | n.d. | - | n.d. | - |
| **Glycosidase (hydrolysis of single sugar residues from non-reducing ends)** | **GH1** | 0.822 | 1 | n.d. | - | 0.074 | 4 | n.d. | - |
|  | **GH2** | 38.604 | 8 | n.d. | - | 0.107 | 6 | n.d. | - |
|  | **GH3** | 3.022 | 2 | n.d. | - | 0.142 0.069 | 6 4 | n.d. | - |
|  | **GH4** | 22.249 144.144 | 6 10 | n.d. | - | 0.019 | 2 | n.d. | - |
|  | **GH29** | n.d. | - | n.d. | - | n.d. | - | n.d. | - |
|  | **GH31** | n.d. | - | n.d. | - | n.d. | - | n.d. | - |
|  | **GH35** | n.d. | - | n.d. | - | n.d. | - | n.d. | - |
|  | **GH36** | n.d. | - | n.d. | - | n.d. | - | n.d. | - |
|  | **GH38** | n.d. | - | n.d. | - | n.d. | - | 0.004 | 1 |
|  | **GH39** | n.d. | - | n.d. | - | n.d. | - | n.d. | - |
|  | **GH42** | n.d. | - | n.d. | - | n.d. | - | n.d. | - |
|  | **GH43** | n.d. | - | n.d. | - | n.d. | - | n.d. | - |
|  | **GH51** | 3.355 | 3 | n.d. | - | 0.113 | 6 | n.d. | - |
|  | **GH62** | n.d. | - | n.d. | - | n.d. | - | n.d. | - |
|  | **GH67** | n.d. | - | n.d. | - | n.d. | - | n.d. | - |
|  | **GH78** | n.d. | - | n.d. | - | n.d. | - | n.d. | - |
|  | **GH120** | n.d. | - | n.d. | - | n.d. | - | n.d. | - |
| **Oligosaccharide phosphorylase** | **GH94** | n.d. | - | n.d. | - | n.d. | - | n.d. | - |
|  | **GH130** | n.d. | - | n.d. | - | n.d. | - | 0.002 | 1 |
