# Supplementary material for: Targeted in situ metatranscriptomics for selected taxa from mesophilic and thermophilic biogas plants
Source: Microb Biotechnol. 2017 Dec 4;11(4):667–79. doi: 10.1111/1751-7915.12982 (PMC6011919; doi:10.1111/1751-7915.12982)
Supplement: Supplementary file 6 — Table S6. Unshortened table of fermentation pathway proteins, their respective Transcript per Million (TPM) values and transcription categories between 0 and 10 for all four genome bins. [file MBT2-11-667-s006.docx]

**Supplementary Table 6:** Unshortened table of fermentation pathway proteins, their respective Transcript per Million (TPM) values and transcription categories between 0 and 10 for all four genome bins.

| **Fermentation Type** | **Fermentation Pathway** | **Enzyme** | **Interpro Number** | ***Thermotogae*** | | ***Fusobacteria*** | | ***Spirochaetes*** | | ***Cloacimonetes*** | |
| --- | --- | --- | --- | --- | --- | --- | --- | --- | --- | --- | --- |
|  |  |  |  | **TPM** | **Category** | **TPM** | **Category** | **TPM** | **Category** | **TPM** | **Category** |
| **Propionic acid fermentation** | **Acrylyl-CoA pathway** | **CoA-transferase (EC 2.8.3.1)** | **IPR003702** | n.d. | - | n.d. | - | n.d. | - | n.d. | - |
|  |  | **Lactoyl-CoA dehydratase (EC 4.2.1.54)** | **IPR010327** | n.d. | - | n.d. | - | n.d. | - | 0.009 | - |
|  |  | **Acyl-CoA dehydrogenase (E.C. 1.3.99.3)** | **IPR034179 IPR034180** | n.d. | - | n.d. | - | n.d. | - | n.d. | - |
|  | **Methylmalonyl-CoA pathway** | **Pyruvate carboxylase (EC 6.4.1.1)** | **IPR005930** | n.d. | - | n.d. | - | n.d. | - | n.d. | - |
|  |  | **Malate dehydrogenase (EC 1.1.1.37)** | **IPR001252 IPR023958 IPR011275** | 25.110 | 7 | 1.308 | 5 | n.d. | - | 0.035 | 7 |
|  |  | **Fumarate hydratase (EC 4.2.1.2)** | **IPR018951 IPR011167** | 1.991 8.148 | 2 4 | 0.982 | 4 | 0.375 | 9 | 0.0104 | 3 |
|  |  | **Fumarate reductase (EC 1.3.5.4)** | **IPR005884** | n.d. | - | n.d. | - | n.d. | - | n.d. | - |
|  |  | **Succinyl-CoA synthetase (EC 6.2.1.4; EC 6.2.1.5)** | **IPR034722 IPR005809 IPR005810** | n.d. | - | n.d. | - | n.d. | - | n.d. | - |
|  |  | **Methylmalonyl-CoA mutase (5.4.99.2)** | **IPR004608 IPR024067** | n.d. | - | 0.021 | 1 | n.d. | - | n.d. | - |
|  |  | **Methylmalonyl-CoA epimerase (EC 5.1.99.1)** | **IPR017515** | 38.108 | 8 | n.d. | - | n.d. | - | n.d. | - |
|  |  | **Methylmalonyl-CoA decarboxylase (EC 4.1.1.41)** | **-** | 24.441 | 7 | n.d. | - | n.d. | - | n.d. | - |
|  |  | **CoA-transferase (EC 2.8.3.1)** | **IPR003702** | n.d. | - | n.d. | - | n.d. | - | n.d. | - |
| **Ethanol fermentation** | | **Pyruvate dehydrogenase (EC 1.2.4.1)** | **IPR017597 IPR027110** | n.d. | - | n.d. | - | n.d. | - | n.d. | - |
|  |  | **Pyruvate decarboxylase (EC 4.1.1.1)** | **-** | n.d. | - | n.d. | - | n.d. | - | n.d. | - |
|  |  | **Alcohol dehydrogenase (EC 1.1.1.1)** | **IPR023921** | 26.691 | 7 | 1.563 | 6 | 0.337 0.303 0.028 | 9 8 2 | 0 | 0 |
| **Formic acid fermentation** | **2,3-Butanediol fermentation** | **Pyruvate formate-lyase (EC 2.3.1.54)** | **IPR005949** | n.d. | - | n.d. | - | n.d. | - | n.d. | - |
|  |  | **Formate Hydrogen Lyase (EC 1.2.1.2)** | **IPR006478 IPR033689** | n.d. | - | n.d. | - | n.d. | - | n.d. | - |
|  |  | **Acetolactate synthase (EC 2.2.6.1)** | **IPR004789 IPR012782 IPR012846 IPR019455** | 57.686 18.144 | 9 6 | 2.784 1.922 | 8 7 | n.d. | - | n.d. | - |
|  |  | **Acetolactate decarboxylase (EC 4.1.1.5)** | **IPR005128** | n.d. | - | n.d. | - | n.d. | - | n.d. | - |
|  |  | **Butanediol dehydrogenase (EC 1.1.1.4)** | **-** | n.d. | - | n.d. | - | n.d. | - | n.d. | - |
|  | **Mixed-acid fermentation** | **Pyruvate carboxylase (EC 6.4.1.1)** | **IPR005930** | n.d. | - | n.d. | - | n.d. | - | n.d. | - |
|  |  | **Malate dehydrogenase (EC 1.1.1.37)** | **IPR001252 IPR023958 IPR011275** | 25.110 | 7 | 1.309 | 5 | n.d. | - | 0.035 | 7 |
|  |  | **Fumarat hydratase (EC 4.2.1.2)** | **IPR018951 IPR011167** | 1.991 8.148 | 2 4 | 0.982 | 4 | 0.375 | 9 | 0.0104 | 3 |
|  |  | **Fumarate reductase (EC 1.3.1.6)** | **IPR027477** | n.d. | - | n.d. | - | n.d. | - | 0.007 | 2 |
|  |  | **Lactate dehydrogenase (EC 1.1.1.28)** | **-** | 19.016 18.631 | 6 6 | n.d. | - | 0.169 0.189 | 7 7 | n.d. | - |
|  |  | **Phosphotransacetylase (EC 2.3.1.8)** | **IPR016475 IPR004614 IPR002505 IPR012147** | n.d. | - | 3.875 | 8 | n.d. | - | 0.021 | 5 |
|  |  | **Acetate kinase (EC 2.7.2.1)** | **IPR000890 IPR004372 IPR023865** | 79.588 | 9 | 4.573 | 8 | 0.136 | 6 | 0.026 | 6 |
| **Butyric acid fermentation** | | **Thiolase (EC 2.3.1.9)** | **-** | n.d. | - | n.d. | - | n.d. | - | n.d. | - |
|  |  | **3-hydroxybutyryl-CoA dehydrogenase (EC 1.1.1.157)** | **-** | n.d. | - | n.d. | - | n.d. | - | n.d. | - |
|  |  | **Crotonase (EC 4.2.1.150)** | **-** | n.d. | - | n.d. | - | n.d. | - | n.d. | - |
|  |  | **Butyryl-CoA dehydrogenase (EC 1.3.8.1)** | **-** | n.d. | - | n.d. | - | n.d. | - | n.d. | - |
|  |  | **Phosphate butyryl transferase (EC 2.3.1.19)** | **IPR014079** | 1.765 | 2 | n.d. | - | n.d. | - | n.d. | - |
|  |  | **Butyrate kinase (2.7.2.7)** | **IPR011245** | 3.494 | 3 | n.d. | - | n.d. | - | 0.029 | 6 |
| **Homoacetogenesis** | | **Pyruvate: ferredoxin oxidoreductase (EC 1.2.7.1)** | **-** | 50.748 49.909 11.622 61.543 | 8 8 5 9 | 14.265 | 10 | 0.826 | 10 | 0.060 0.050 | 8 8 |
|  |  | **Phosphotransacetylase (EC 2.3.1.8)** | **IPR016475 IPR004614 IPR002505 IPR012147** | n.d. | - | 3.874 | 8 | n.d. | - | n.d. | - |
|  |  | **Acetate kinase (EC 2.7.2.1)** | **IPR000890 IPR004372 IPR023865** | 79.588 | 9 | 4.573 | 8 | 0.136 | 6 | 0.026 | 6 |
| **Lactic acid fermentation** | **Homolactic acid fermentation** | **Glucose-6-phosphate isomerase (EC 5.3.1.9)** | **IPR001672 IPR010551 IPR016758 IPR018189 IPR023096** | 22.717 | 6 | 5.772 | 9 | 0.520 | 9 | 0.011 | 3 |
|  |  | **6-phospho-fructokinase (EC 2.7.1.11)** | **IPR000023 IPR012003 IPR012004 IPR012828 IPR015912 IPR022953** | 74.683 | 9 | 7.921 | 9 | n.d. | - | 0.057 | 8 |
|  |  | **Fructose-bisphosphate aldolase (EC 4.1.2.13)** | **IPR023014 IPR000741 IPR011289 IPR029768** | 152.525 | 10 | 15.580 | 10 | 1.015 0.111 | 10 5 | n.d. | - |
|  |  | **Triosephosphate isomerase (5.3.1.1)** | **IPR000652 IPR020861 IPR022891 IPR022896** | n.d. | - | n.d. | - | n.d. | - | n.d. | - |
|  |  | **Lactate dehydrogenase (EC 1.1.1.28)** | **-** | 19.015 18.635 | 6 6 | n.d. | - | 0.169 0.189 | 7 7 | n.d. | - |
|  | **Heterolactic acid fermentation** | **Hexokinase (EC 2.7.1.1)** | **IPR001312 IPR019807 IPR022672 IPR022673** | n.d. | - | n.d. | - | 0.115 | 5 | n.d. | - |
|  |  | **Glucose-6 phosphate dehydrogenase (EC 1.1.1.49)** | **IPR001282 IPR019796 IPR022674 IPR022675** | 18.460 | 6 | n.d. | - | 0.128 | 6 | n.d. | - |
|  |  | **6-phosphogluconolactonase (EC 3.1.1.31)** | **IPR022528** | n.d. | - | n.d. | - | 0.346 | 9 | n.d. | - |
|  |  | **Phosphogluconate dehydrogenase (EC 1.1.1.44)** | **IPR006184 IPR006183 IPR006114 IPR006113** | 6.055 | 3 | n.d. | - | 0.095 | 5 | n.d. | - |
|  |  | **Ribulose-phosphate 3-epimerase (EC 5.1.3.1)** | **IPR000056 IPR026019** | 28.915 | 7 | 2.740 | 8 | 0.578 | 9 | 0.012 | 3 |
|  |  | **Xylulose-5-phosphate phosphoketolase (EC 4.1.2.9)** | **-** | n.d. | - | n.d. | - | n.d. | - | n.d. | - |
|  |  | **Acyl-phosphatase (EC 3.6.1.7)** | **IPR001792 IPR017968 IPR020456 IPR028627** | 15.719 | 5 | n.d. | - | n.d. | - | 0.021 | 5 |
|  |  | **Acetate kinase (EC 2.7.2.1)** | **IPR000890 IPR004372 IPR023865** | 79.588 | 9 | 4.573 | 8 | 0.136 | 6 | 0.026 | 6 |
|  |  | **Phosphotransacetylase (EC 2.3.1.8)** | **IPR016475 IPR004614 IPR002505 IPR012147** | n.d. | - | 3.874 | 8 | n.d. | - | n.d. | - |
|  |  | **Acetaldehyde dehydrogenase (EC 1.2.1.10)** | **IPR003361 IPR015426** | n.d. | - | n.d. | - | n.d. | - | n.d. | - |
|  |  | **Alcohol dehydrogenase (EC 1.1.1.1)** | **IPR023921** | 26.691 | 7 | 1.563 | 6 | 0.337 0.303 0.028 | 9 8 2 | 0 | 0 |
