# Supplementary material for: Targeted in situ metatranscriptomics for selected taxa from mesophilic and thermophilic biogas plants
Source: Microb Biotechnol. 2017 Dec 4;11(4):667–79. doi: 10.1111/1751-7915.12982 (PMC6011919; doi:10.1111/1751-7915.12982)
Supplement: Supplementary file 7 — Table S7. Unshortened table of possibly syntrophy associated proteins, their respective Transcript per Million (TPM) values and transcription categories between 0 and 10 for all four genome bins. [file MBT2-11-667-s007.docx]

**Supplementary Table 7:** Unshortened table of possibly syntrophy associated proteins, their respective Transcript per Million (TPM) values and transcription categories between 0 and 10 for all four genome bins.

| **Enzyme** | **Subunit** | **Interpro Number** | ***Thermotogae*** | | ***Fusobacteria*** | | ***Spirochaetes*** | | ***Cloacimonetes*** | |
| --- | --- | --- | --- | --- | --- | --- | --- | --- | --- | --- |
|  |  |  | **TPM** | **Category** | **TPM** | **Category** | **TPM** | **Category** | **TPM** | **Category** |
| **Capsule synthesis protein, CapA** |  | **IPR019079** | n.d. | - | n.d. | - | n.d. | - | 0.04 | 7 |
| **Cell cycle, FtsW n.d. RodA n.d. SpoVE** |  | **IPR018365** | n.d. | - | 0.42 0.64 0.42 | 2 3 2 | n.d. | - | n.d. | - |
| **Ribonuclease P, conserved site** |  | **IPR020539** | n.d. | - | n.d. | - | n.d. | - | 0.12 | 9 |
| **Cytoplasmic FDH** | **Alpha** | **IPR027467** | n.d. | - | n.d. | - | n.d. | - | n.d. | - |
|  |  | **IPR006655** | n.d. | - | n.d. | - | n.d. | - | n.d. | - |
|  |  | **IPR006478** | n.d. | - | n.d. | - | n.d. | - | n.d. | - |
|  | **NUO 51kDa** | **IPR019575** | n.d. | - | n.d. | - | 0.054 | 3 | n.d. | - |
|  |  | **IPR001949** | 79.06 | 9 | 0.36 | 8 | 0.054 | 3 | 0.02 0.02 | 5 5 |
| **Extra–cytopl. FDH** | **Alpha** | **IPR006443** | 79.06 | 9 | 0.36 | 8 | n.d. | - | 0.02 | 5 |
| **Formate Transporter** |  | **IPR000292** | n.d. | - | n.d. | - | n.d. | - | n.d. | - |
|  |  | **IPR024002** | n.d. | - | n.d. | - | n.d. | - | n.d. | - |
| **FeFe–hydrogenase** | **Alpha** | **IPR004108** | n.d. | - | n.d. | - | 0.075 | 4 | n.d. | - |
|  |  | **IPR009016** | 110.75 | 9 | 2.07 9.26 | 7 10 | 0.075 | 4 | 0.05 | 8 |
|  |  | **IPR003149** | 110.75 | 9 | 2.07 9.27 | 7 10 | 0.075 | 4 | 0.05 | 8 |
|  |  | **IPR013352** | 110.75 | 9 | 9.27 | 10 | 0.075 | 4 | 0.05 | 8 |
| **NiFe–hydrogenase** |  | **IPR001501** | 110.75 | 9 | 9.27 | 10 | n.d. | - | 0.05 | 8 |
|  |  | **IPR018194** | n.d. | - | n.d. | - | n.d. | - | n.d. | - |
| **Rnf complex** | **RnfB** | **IPR007202** | n.d. | - | n.d. | - | n.d. | - | n.d. | - |
|  |  | **IPR010207** | 798.79 | 10 | 2.07 2.68 | 7 8 | 0.25 | 8 | 0.02 | 5 |
|  | **RnfC** | **IPR026902** | 798.79 | 10 | 2.68 | 8 | 0.34 | 9 | 0.02 | 5 |
|  |  | **IPR010208** | 14.37 | 5 | 4.01 | 8 | 0.34 | 9 | 0.01 | 2 |
|  | **RnfD** | **IPR004338** | 14.37 | 5 | 4.01 | 8 | 3.24 | 10 | 0.01 | 2 |
|  |  | **IPR011303** | 7.63 | 4 | 0.93 | 4 | 0.39 | 9 | 0.01 | 2 |
|  | **RnfG** | **IPR007329** | 7.63 | 4 | 0.93 | 4 | n.d. | - | 0.01 | 2 |
| **Ech complex** | **EchA** | **IPR001750** | 1100.12 162.44 | 10 10 | 12.01 | 10 | n.d. | - | 0.01 0.01 | 2 2 |
|  |  | **IPR001516** | n.d. | - | n.d. | - | n.d. | - | n.d. | - |
|  | **EchB** | **IPR001694** | n.d. | - | n.d. | - | n.d. | - | n.d. | - |
|  | **EchC** | **IPR006137** | n.d. | - | n.d. | - | n.d. | - | n.d. | - |
|  | **EchD** | **IPR001268** | n.d. | - | n.d. | - | n.d. | - | n.d. | - |
|  |  | **IPR012179** | n.d. | - | n.d. | - | n.d. | - | n.d. | - |
|  | **EchE** | **IPR001135** | n.d. | - | n.d. | - | n.d. | - | n.d. | - |
| **Etf Alpha** | **-** | **IPR014731** | n.d. | - | n.d. | - | 0.41 | 9 | n.d. | - |
| **Etf Beta** | **-** | **IPR012255** | n.d. | - | n.d. | - | 1.19 | 10 | 0.03 | 6 |
| **Bcd** |  | **IPR006089** | n.d. | - | n.d. | - | n.d. | - | 0.02 | 5 |
|  |  | **IPR009075** | n.d. | - | n.d. | - | n.d. | - | 0.05 | 8 |
|  |  | **IPR006092** | n.d. | - | n.d. | - | n.d. | - | 0.05 | 8 |
|  |  | **IPR006091** | n.d. | - | n.d. | - | n.d. | - | n.d. | - |
|  |  | **IPR013786** | n.d. | - | n.d. | - | n.d. | - | 0.05 | 8 |
|  |  | **IPR009100** | n.d. | - | n.d. | - | n.d. | - | 0.05 | 8 |
| **DUF224** |  | **IPR003816** | n.d. | - | n.d. | - | n.d. | - | 0.05 | 8 |
|  |  | **IPR004017** | n.d. | - | n.d. | - | n.d. | - | n.d. | - |
|  |  | **IPR023234** | n.d. | - | n.d. | - | n.d. | - | n.d. | - |
| **Cytochrome** | **c** | **IPR023155** | n.d. | - | n.d. | - | n.d. | - | n.d. | - |
|  |  | **IPR024673** | n.d. | - | n.d. | - | n.d. | - | n.d. | - |
|  | **cIII** | **IPR020942** | n.d. | - | n.d. | - | n.d. | - | n.d. | - |
|  |  | **IPR002322** | n.d. | - | n.d. | - | n.d. | - | n.d. | - |
|  | **b561** | **IPR016174** | n.d. | - | n.d. | - | n.d. | - | n.d. | - |
|  |  | **IPR000516** | n.d. | - | n.d. | - | n.d. | - | n.d. | - |
|  | **b5** | **IPR001199** | n.d. | - | n.d. | - | n.d. | - | n.d. | - |
